# Concentration strategies for spiked and naturally present biomarkers in non-invasively collected first-void urine

**DOI:** 10.1186/s40001-024-01719-5

**Published:** 2024-02-17

**Authors:** Laura Téblick, Marijana Lipovac, F. Ricardo Burdier, Annemie De Smet, Margo Bell, Eef van den Borst, Veerle Matheeussen, Alex Vorsters

**Affiliations:** 1 Centre for the Evaluation of Vaccination (CEV), Vaccine & Infectious Disease Institute (VAXINFECTIO), Faculty of Medicine and Health Sciences, University of Antwerp, 2610 Wilrijk-Antwerp, Belgium; 2 Centre of Medical Genetics, Faculty of Medicine and Health Sciences, University of Antwerp and Antwerp University Hospital, 2650 Edegem, Belgium; 3 Department of Microbiology, Antwerp University Hospital (UZA), 2650 Edegem-Antwerp, Belgium; 4 Department of Medical Microbiology (LMM), Vaccine & Infectious Disease Institute (VAXINFECTIO), Faculty of Medicine and Health Sciences, University of Antwerp, 2610 Wilrijk-Antwerp, Belgium; 5 Department of Medical Biochemistry, Faculty of Pharmaceutical, Biomedical and Veterinary Sciences, University of Antwerp, 2610 Wilrijk-Antwerp, Belgium

**Keywords:** First-void urine, FVU, Concentration, Human papillomavirus, Biomarkers

## Abstract

**Background:**

First-void urine (FVU) provides a non-invasive method for collecting a wide range of biomarkers found in genital tract secretions. To optimize biomarker collection in FVU, this study investigated the impact of naturally present and supplemented precipitating agents: uromodulin (UMOD) and polyethylene glycol (PEG), on the concentration of human papillomavirus (HPV) pseudovirions (PsV), cell-free DNA (cfDNA), and cellular genomic DNA (gDNA) through centrifugation.

**Methods:**

FVU samples from ten healthy female volunteers, along with a control sample, were spiked with seal herpesvirus 1 (PhHV-1) DNA, HPV16 plasmid DNA, and HPV16 PsV with an enhanced green fluorescent protein (EGFP) reporter. The samples were subjected to various concentration protocols involving PEG precipitation, low-speed centrifugation (5 min at 1000×*g*), and medium-speed centrifugation (1 h at 3000×*g*). Subsequently, quantitative PCR (qPCR) was used to assess cellular and cell-free glyceraldehyde-3-phosphate dehydrogenase (GAPDH) DNA, cell-free PhHV-1 and HPV16 DNA, and PsV (EGFP) DNA. In addition, UMOD levels were measured.

**Results:**

The findings revealed that PEG significantly increased the concentration of cfDNA and gDNA in the pellet after centrifugation, with the most pronounced effect observed for cfDNA. Moreover, low-speed centrifugation without PEG effectively depleted cellular gDNA while preserving cfDNA in the supernatants. Pseudovirions were consistently pelleted, even with low-speed centrifugation, and a positive but not significant effect of PEG on PsV (EGFP) DNA yield in the pellet was observed. Additionally, a significant correlation was observed between UMOD and GAPDH, HPV16, and PsV (EGFP) DNA quantities in the pellet. Furthermore, large variations among the FVU samples were observed.

**Conclusions:**

With this study, we provide novel insights into how various biomarker precipitation protocols, including both the properties of FVU and the use of PEG as a precipitating agent, influence the concentration of cfDNA, cellular gDNA, and pseudovirions.

**Supplementary Information:**

The online version contains supplementary material available at 10.1186/s40001-024-01719-5.

## Introduction

Secretions originating from the female genital tract (FGT), which include cervical mucus, proteins, pathogens, and other biomarkers, contain essential biological information [1, 2]. These secretions can be captured by the first stream of urine, known as first-void urine (FVU) [3, 4]. FVU is a non-invasive and convenient sample that can be collected by individuals from home, making it a practical and accessible option for population-level studies [5–7].

For human papillomavirus (HPV) based studies, FVU sampling has demonstrated added value as it allows for the detection of virological (HPV DNA), diagnostic (methylation markers), and immunological (HPV-type specific antibodies) endpoints [8–13]. Given that cervical cancer, primarily caused by HPV, remains the fourth most common cancer in women worldwide, large epidemiological trials are warranted, and the impact and effect of vaccinating adults needs to be elucidated [14, 15]. Using FVU as a source of local biomarkers at the site of infection simplifies studies in this context. In addition to DNA and antibodies, intact HPV virions are hypothesized to be present in FVU, enabling the investigation of antibody–virion interactions [14, 16]. Moreover, FVU sampling holds potential beyond HPV-related research, containing valuable information on sexually transmitted infections (STIs) and serving as a biomarker source for cancer-related research [17–22].

FVU captures proteins, DNA, metabolites, viral particles, bacteria, immune cells and (debris of) exfoliating cells [13, 23–25]. However, targeting the secretions of interest can be challenging as certain biomarkers are present in only small amounts. Therefore, concentrating the sample is essential for identifying all the information of interest [26], and various methods, including centrifugation/precipitation, have been explored for this purpose [27, 28].

Urine contains a variety of proteins that may influence the detection and concentration of biomarkers of interest, with uromodulin (UMOD), also called Tamm–Horsfall protein (THP), being a notable example [29, 30]. UMOD is produced primarily by the kidneys, plays a role in kidney function, and is considered the most abundant protein in urine [31]. The UMOD in urine self-assembles into large, linear polymers, known as uromodulin filaments, which may interact with other proteins and biomarkers in urine. This makes it interesting to study the effect of UMOD on biomarker precipitation [32]. Another potential precipitating agent that is not present in urine is polyethylene glycol (PEG), which induces the formation of protein aggregates by altering the solution conditions, leading to changes in protein solubility and promoting protein precipitation [33–35]. However, the effect of PEG and UMOD on the precipitation of (DNA) biomarkers in FVU has not yet been investigated.

In addition to the complexity introduced by sample variations, different types of DNA in FVU may react, bind, and concentrate in various ways. While cellular genomic DNA (gDNA) or large proteins and cellular debris present in FVU may be more prone to precipitate and interact with internal or external precipitating agents, cell-free DNA (cfDNA), which is smaller in size, may remain unaffected. Furthermore, DNA captured in viral particles is larger in size than cell-free DNA (cfDNA) and might also affect precipitation. Thus, to evaluate the effect of specific parameters on the precipitation and concentration of biomarkers, it is essential to investigate different types of DNA.

In this study, we evaluated the effects of different concentration protocols on the precipitation of pseudovirion (PsV) encapsulated DNA (enhanced green fluorescent protein, EGFP), spiked cfDNA (seal herpesvirus 1 (PhHV-1) and HPV16 plasmid), and a combination of human cfDNA and gDNA (glyceraldehyde-3-phosphate dehydrogenase, GAPDH). Furthermore, the effects of precipitating agents UMOD and PEG on the concentration protocols were evaluated. Consequently, the results of this study provide essential information on the optimal precipitation protocols for the detection and concentration of different DNA biomarkers and PsV from FVU.

## Materials and methods

### Sample collection

We collected FVU samples from ten healthy female volunteers at the Centre for the Evaluation of Vaccination, University of Antwerp, Belgium. Each female volunteer collected one first-void urine sample using a 20 ml Colli-pee first-void urine collection device (Novosanis, Belgium) prefilled with 1/3 urine conservation medium (UCM), resulting in the collection of 13.67 ml FVU directly preserved in 6.33 ml of UCM. The samples were divided into 1-ml aliquots and immediately stored at − 80 °C before further analysis. We obtained informed consent from all volunteers, and the data and samples were coded to ensure participant privacy. This study was approved by the Institutional Review Board of UZA/University of Antwerp (B300201734129).

### Sample processing

**Fig. 1:**
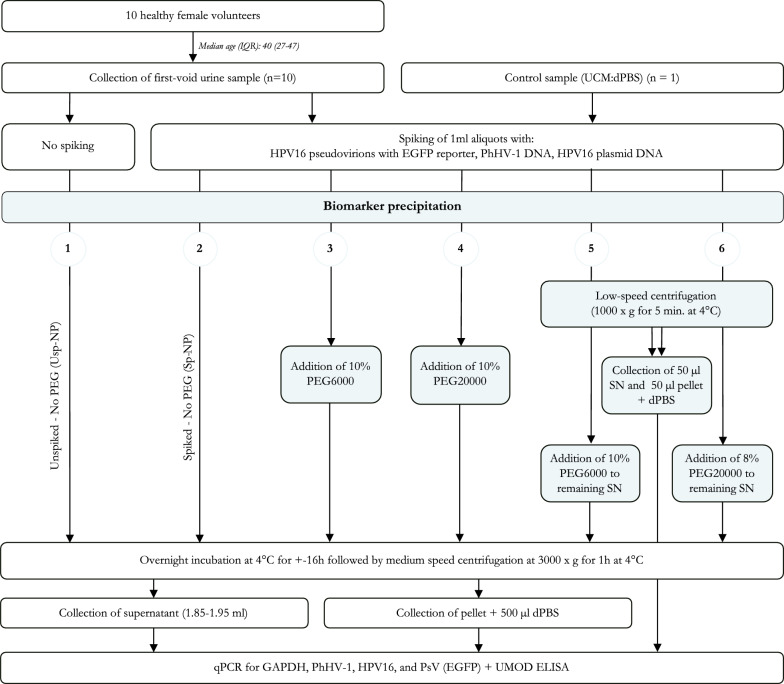
Schematic overview. First-void urine (FVU) samples were collected from 10 female volunteers. Aliquots of 1 ml of the FVU and the control sample were spiked with HPV16 PsV, PhHV-1 DNA, and HPV16 plasmid DNA. Also, one aliquot was not spiked. All the samples underwent biomarker precipitation using six different protocols; (1) precipitation of unspiked samples without PEG (Usp-NP), (2) precipitation of spiked samples without PEG (Sp-NP), (3) precipitation using 10% (w/v) PEG6000, (4) precipitation using 8% (w/v) PEG20000, (5) low-speed centrifugation followed by precipitation of the supernatant using 10% (w/v) PEG6000, and (6) low-speed centrifugation followed by precipitation of the supernatant using 8% (w/v) PEG20000. Supernatants and pellets were collected for all purifications and qPCR was performed for GAPDH, PhHV-1, HPV16, and PsV (EGFP). In addition, ELISA for UMOD was performed

Aliquots of 1 ml FVU and control samples, consisting of 0.33 ml UCM and 0.67 ml dPBS, were used. For each ID and the control samples, six aliquots were spiked with (I) 0.74 ng/µl HPV16 PsV, (II) 16.7 µl of a 1:1000 dilution of PhHV-1 DNA, and (III) 5 × 10^6^ copies/ml HPV16 plasmid DNA. HPV16 PsV were produced based on the protocol of Buck et al. with enhanced green fluorescent protein (EGFP) serving as a reporter plasmid [36]. A PhHV-1 stock was created by extracting DNA from 200 µl MEM culture medium from a PhHV-1 infected Crandell Rees Feline kidney cell line using NucliSENS® EasyMag® (bioMérieux, off-board lysis protocol) and elution in 100 µl. Commercially available plasmids containing the HPV16 genome were used (Clonit, Milan, Italy). One 1 ml aliquot of each ID and the control sample was not spiked with DNA. In total, six aliquots of each sample were used for sample concentration based on the different protocols described below (Fig. 1), and one aliquot (spiked) was used for baseline analysis.

#### Low-speed centrifugation

Two aliquots of each sample were centrifuged for 5 min at 1000×*g* at 4 °C (LSC). After centrifugation, the supernatant was removed, whereof 50 µl was collected for direct DNA extraction and the remaining volume of supernatant, approximately 900 µl, was used for PEG precipitation. The pellet was resuspended in dPBS to reach a final volume of 50 µl and was stored for direct DNA extraction.

#### PEG precipitation

##### PEG stock preparation

We prepared a 50 ml stock of 20% (w/v) PEG6000 solution by adding 10 g of PEG6000 and 0.5 M NaCl to 50 ml dH_2_O. A 50 ml stock of 16% (w/v) PEG20000 was prepared by adding 8 g of PEG20000 and 0.5 M NaCl to 50 ml dH_2_O. Both stocks were stored for a maximum of 1 week at 4 °C until use.

##### Medium-speed centrifugation

A 1-ml aliquot and one aliquot of 900 µl supernatant after low-speed centrifugation (2.2.1) were diluted with an equal amount of 20% (w/v) PEG6000 solution to obtain a final concentration of 10% (w/v) PEG6000. The same procedure was used for the 16% (w/v) PEG20000 solution, which was added in equal amounts to a 1-ml aliquot and one aliquot of 900 µl supernatant after low-speed centrifugation (2.2.1) to obtain a final concentration of 8% PEG20000. The samples were incubated with PEG at 4 °C for 16 h. Additionally, one 1-ml aliquot with spiked DNA (Spiked—No PEG, Sp-NP) and one without (Unspiked—No PEG, Usp-NP), were incubated at 4 °C for 16 h without the addition of PEG. After incubation, all 6 aliquots were centrifuged at 3000×*g* for 1 h at 4 °C. The supernatant was collected, and 50 µl was used for DNA extraction. The pellet was resuspended in dPBS to reach a final volume of 500 µl.

### DNA extraction and qPCR

In this experiment, various purifications (both supernatant and pellet) and baseline aliquots were subjected to quantitative PCR (qPCR) to detect specific biomarker DNA [GAPDH, PhHV-1, HPV16, and PsV (EGFP)]. For all the samples and purifications, 50 µl was added to 2 ml of NucliSENS Lysis Buffer (bioMérieux Benelux, Schaarbeek, Belgium). DNA extraction was performed using NucliSENS® EasyMag® (bioMérieux) with an off-board lysis generic protocol and elution in 55 µl. The LightCycler480 Real-Time PCR instrument was used (Roche Diagnostics, Machelen, Belgium) following the protocol described by Vorsters et al. [37]. Briefly, for GAPDH and HPV16, a 20-µl portion of the PCR mixture containing 1× LightCycler® 480 Probes Master (Roche Applied Science, Belgium), 0.5 µM of each primer, 0.1 µM of the probe, and 5 µl of DNA solution was loaded into the LightCycler. For PsV (EGFP), qPCR was performed on 20 µl of a mixture containing 1× LightCycler® 480 Probes Master (Roche Applied Science, Belgium), 0.25 µM forward primer, 0.25 µM reverse primer, 0.2 µM probe, and 5 µl DNA solution. For PhHV-1, qPCR was performed on 20 µl of a mixture containing 1× LightCycler® 480 Probes Master (Roche Applied Science, Belgium), 0.05 µM forward primer, 0.2 µM reverse primer, 0.1 µM probe, and 5 µl sample. The GAPDH, PhHV-1, and HPV16 plasmid primers and probes used have been published previously [38–40]. For EGFP, the following primers were used; EGFP-F: CACTACCTGAGCACCCAGTC, EGFP-R: CACGAACTCCAGCAGGACCATG, and EGFP-TM: F-CGCTTCTCGTTGGGGTCTTTGCT—Q. The lengths of the amplification products were 156 bp for GAPDH, 89 bp for PhHV-1, 81 bp for HPV16, and 58 bp for EGFP. The thermal cycling protocol consisted of the following steps: initial activation of DNA polymerase at 95 °C for 10 min; 45 cycles of denaturation at 95 °C for 10 s; and annealing at 60 °C for 15 s. To ensure reproducibility, both positive and negative controls were included in each run. DNA concentrations for each parameter were calculated based on the Cq values. For PsV (EGFP) and PhHV-1, the results are reported as arbitrary copies/ml as the concentration of the used standards were unknown. For GAPDH, results were reported as ng/ml, and for HPV16 as copies/ml. The following standard curves were used: EGFP: *y* = − 0.2997 * *x* + 14.496, PhHV-1: *y* = − 0.301 * *x* + 13.614, GAPDH: − 3.657 * *x* + 41.60, HPV16: *y* = − 0.2997 * *x* + 12.816, with *x* = log(concentration) and *y* = Cq. All standard curves had an *R*^2^ close to one (> 0.98). Results are converged to quantity (ng, copies, or arbitrary copies) of DNA for further analysis, taking the volumes and dilutions into account.

### UMOD ELISA

UMOD protein concentrations were measured using a Uromodulin Huma ELISA kit (BioVendor, Czech Republic) according to the manufacturer’s instructions. Before analysis, baseline samples were centrifuged at 3820×*g* for 10 min at 20 °C using an Amicon Ultra‐4 50K filter device (Merck Millipore). 1× dPBS (Gibco) was added to the concentrate retained on the filter to reach a final volume of 0.5 ml. All the other arms/fractions were not further processed. The dilutions to be tested were optimized for each sample type, and we selected 1:2000 for Amicon-filtered baseline samples, 1:1000 for supernatant and pellet samples. The absorbance at 450 nm and 630 nm was measured using the Victor Nivo multimode plate reader (Revvity, Belgium). The reference signal at 630 nm was subtracted from the signal at 450 nm for each well, and this measurement was used to calculate the concentration using four-parameter logistic (4PL) curve fitting. Results are calculated as ng/ml UMOD and converged to UMOD quantities (ng) in that specific arm and volume.

### Statistical analysis

All the statistical analyses were performed using R statistical software version 4.2.2. All the data did not meet the normality assumption (Shapiro–Wilk test), and therefore the non-parametric Wilcoxon signed-rank test was applied to check for significant differences between arms and fractions. Statistical significance was determined as *p*-adjusted < 0.05, and the Holm–Bonferroni method was used for adjusting the *p*-values. To explore potential correlations between UMOD and DNA amount in certain fractions, Spearman’s rank correlations were calculated.

## Results

For this study, FVU samples from ten female volunteers and a control sample were spiked with (I) seal herpesvirus 1 (PhHV-1) DNA, which is a nonhuman viral control [26, 39]; (II) HPV16 plasmid DNA, which consists of small, circular pieces of double-stranded DNA; and (III) HPV16 pseudovirions consisting of EGFP reporter DNA to compare different concentration protocols. In addition to the spiked DNA, human cell-free and genomic cellular GAPDH present in FVU was used as an additional parameter. Since the control sample did not contain human DNA, this sample was excluded for GAPDH analysis.

### Effect of PEG on concentrating DNA

**Fig. 2:**
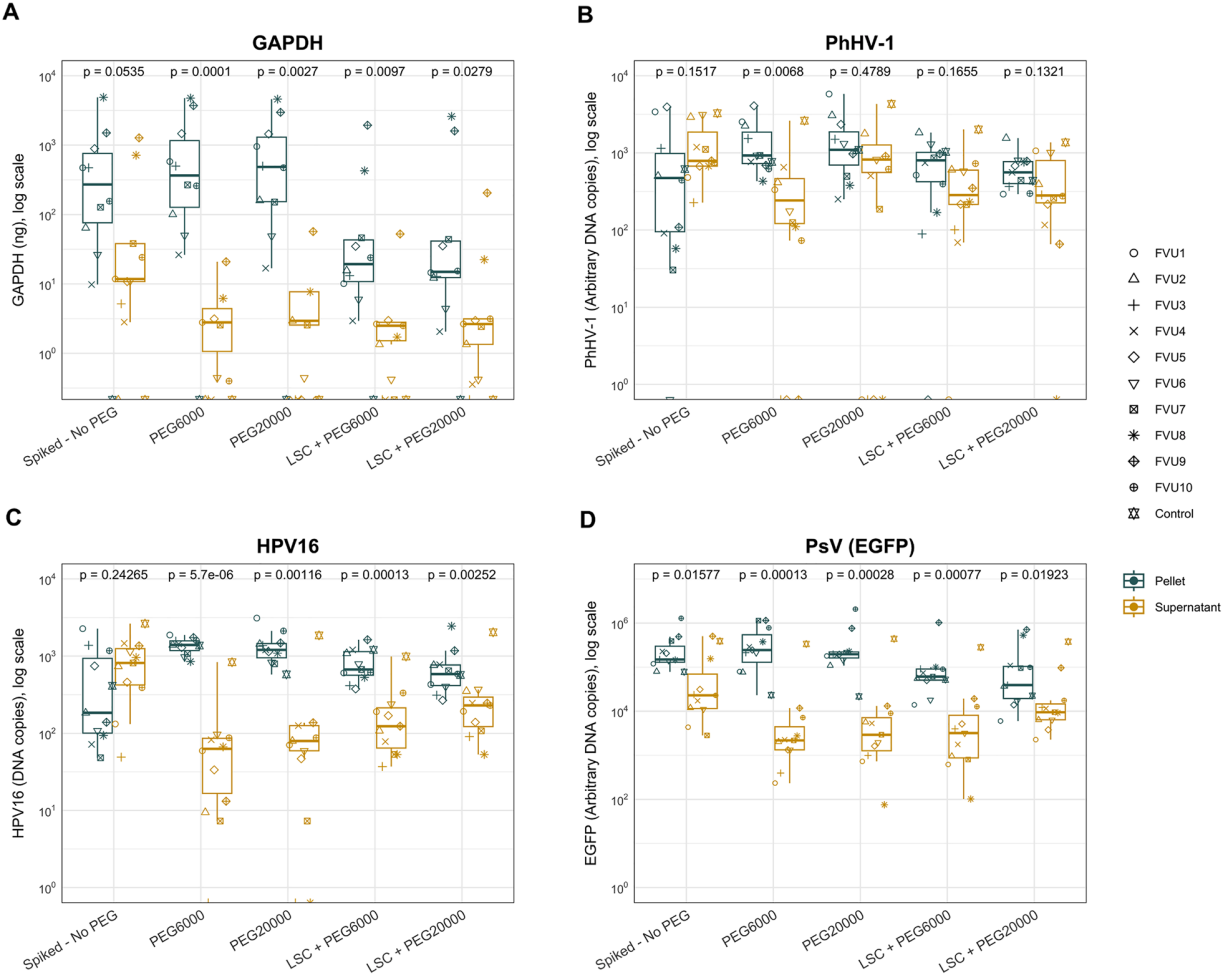
Boxplots representing the amount of **A** GAPDH; **B** PhHV-1; **C** HPV16; and **D** PsV (EGFP) DNA in the respective samples. Results are presented as nanograms (ng), arbitrary DNA copies, and DNA copies. The spiked—No PEG arm did not undergo PEG treatment, whereas the other arms were incubated with 10% (w/v) PEG6000 or 8% (w/v) PEG20000 for approximately 16 h at 4 °C. All the samples were centrifuged at 3000×*g* at 4 °C for 1 h and the low-speed centrifugation (LSC) samples were subjected to low-speed centrifugation without PEG addition at 1000×*g* for 5 min before subsequent PEG precipitation. Significant differences between the pellet and supernatant for each arm are presented in the figure

For each supernatant, pellet and baseline sample, DNA extraction was performed, and the concentration was determined or calculated using standard curves. To evaluate the effect of PEG and centrifugation on the concentration of specific DNA biomarkers, we calculated the amount of biomarker DNA in that specific part/sample, taking the volumes and dilutions into account. Based on pilot experiments, we compared two different PEG conditions on the pelleting of several biomarkers and compared this to the yields without PEG addition. Furthermore, we evaluated the effect of a low-speed centrifugation step on the concentration of DNA. The amount of DNA for these arms and fractions is presented as boxplots (Fig. 2), and median (IQR) quantities are summarized (Table 1). For the Sp-NP arm, PhHV-1 and HPV16 plasmid DNA was most abundant in the supernatant, and PsV (EGFP) and GAPDH were most abundant in the pellet. Both PEG conditions had a positive effect on the yield of GAPDH, PhHV-1, and HPV16 DNA in the pellet after centrifuging for 1 h at 3000×*g*, as the amount of pelleted DNA was significantly higher after PEG addition (*p* ≤ 0.04). For PsV (EGFP), the addition of both PEG6000 and PEG20000 caused an increase in the quantity of DNA in the pellet, but this change was not statistically significant (*p* ≥ 0.17). Additionally, when PEG was used, the amount of DNA in the pellet was significantly higher than in supernatant (*p* ≤ 0.0068) for all arms except for the PEG20000 arm on PhHV-1 (*p* = 0.48). The difference between the pellet and supernatant for the samples that were centrifuged at low-speed for 5 min at 1000×*g* before PEG precipitation was significant for GAPDH, HPV16, and PsV (EGFP) (*p* ≤ 0.028) but not for PhHV-1 (*p* ≥ 0.13) DNA. Overall DNA quantities were lower for all the arms including a low-speed centrifugation step (Table 1). Altogether, the difference between the amount of DNA in the pellet and the supernatant was most significant for the PEG6000 precipitation arms (*p* ≤ 0.0068), although there was no significant difference in the concentration of DNA in the pellet between PEG6000 and PEG20000 (*p* ≥ 0.28).

**Table 1:** Overview of DNA and UMOD quantities (median, IQR) in different fractions of various arms

	GAPDH (ng)	PhHV-1 (arbitrary copies)	HPV16 (DNA copies)	PsV (EGFP) (arbitrary copies)	UMOD (ng)
**No PEG**					
Baseline	311 (76–807)	1709 (1380–2120)	1330 (1110–1635)	189,500 (125,000–404500)	7.62 (4.71–10.84)
Low-speed centrifugation					
Pellet	193 (41–388)	109 (52–638)	78 (58–255)	74,000 (48,138–107,125)	
Supernatant	23 (12–55)	1131 (961–1473)	979 (861–1223)	50,730 (32,728–130,388)	
Unspiked—no PEG					
Pellet	193 (51–756)	0 (0–0)	0 (0–0)	0 (0–0)	
Supernatant	14 (12–42)	0 (0–0)	0 (0–0)	0 (0–0)	
Spiked—no PEG					
Pellet	314 (80–786)	277 (66–984)	162 (98–1068)	177,500 (142,250–350000)	13.62 (8.19–17.98)
Supernatant	11 (7–35)	768 (674–1169)	766 (408–1102)	20,093 (11,329–29,403)	2.01 (1.80–2.64)
**PEG**					
PEG6000					
Pellet	384 (104–1244)	928 (709- 2064)	1415 (133–1584)	266,750 (211,750–697125)	12.69 (4.36–21.33)
Supernatant	1 (0–3)	118 (18–294)	47 (10–79)	2106 (1331–2642)	2.36 (1.49–3.35)
PEG20000					
Pellet	486 (154–1323)	1188 (617–2130)	1290 (1103–1468)	197,500 (180,375–224375)	5.43 (3.49–19.35)
Supernatant	0 (0–3)	346 (0–768)	65 (17–86)	2447 (1149–5572)	2.94 (1.98–4.00)
Low-speed centrifugation + PEG6000					
Pellet	20 (11–43)	630 (226–943)	643 (549–1011)	67,500 (51,063–90750)	0.3 (0.19–0.36)
Supernatant	2 (0–3)	224 (129–522)	116 (59–185)	2485 (841–4870)	1.80 (1.30–2.09)
Low-speed centrifugation + PEG20000					
Pellet	15 (12–42)	620 (386–775)	615 (410–771)	67,100 (21,750–106500)	0.23 (0.16–0.31)
Supernatant	3 (1–3)	226 (141–366)	211 (115–246)	9380 (6387–12,034)	2.48 (1.21–3.14)

No PEG was added to the baseline samples, the low-speed centrifugation (5 min centrifugation at 1000×*g*), and the unspiked and spiked—no PEG arms (1 h centrifugation at 3000×*g*). Two different PEG precipitation protocols were evaluated separately or in combination with the low-speed centrifugation step

### Effect of centrifugation protocol on concentrating DNA

**Fig. 3:**
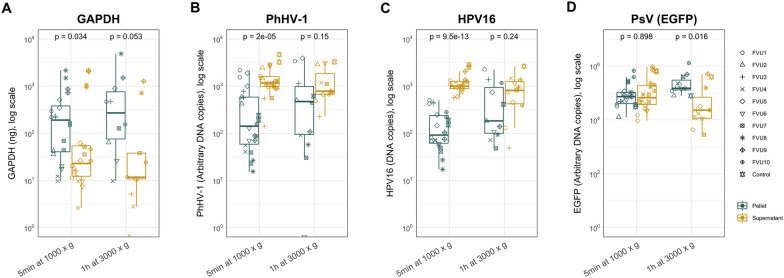
Boxplots representing the amount of **A** GAPDH; **B** PhHV-1; **C** HPV16; and **D** PsV (EGFP) DNA in the respective samples after various centrifugation steps. Results are presented as ng, arbitrary copies, and DNA copies. All 1 ml samples were spiked with the same amount of PhHV-1 DNA, HPV16 plasmid DNA and HPV16 PsV (EGFP). The samples were centrifuged at 1000×*g* at 4 °C for 5 min or at 3000×*g* at 4 °C for 1 h. Centrifugation at 1000×*g* was performed before both PEG precipitation protocols, resulting in two data points per ID for this arm. Significant differences between the pellet and supernatant for centrifugation conditions are presented in the figure

We compared the effects of two different centrifugation protocols on the presence of DNA in each fraction (Fig. 3). Samples were centrifuged at 1000×*g* for 5 min (low-speed centrifugation) or at 3000×*g* for 1 h. Since both centrifugation protocols were performed for both PEG precipitation protocols, two data points per ID were included for these arms. For PhHV-1 and HPV16, DNA was more abundant in the supernatant for both conditions, whereas for GAPDH and EGFP (PsV), the pellet contained the majority of the DNA. For all biomarkers, increasing the centrifugal force and centrifugation time resulted in higher DNA quantities in the pellet. This increase was significant for GAPDH, HPV16, and EGFP (PsV) (*p* ≤ 0.0098) but not for PhHV-1 (*p* ≥ 0.064).

### The effect of UMOD

**Fig. 4:**
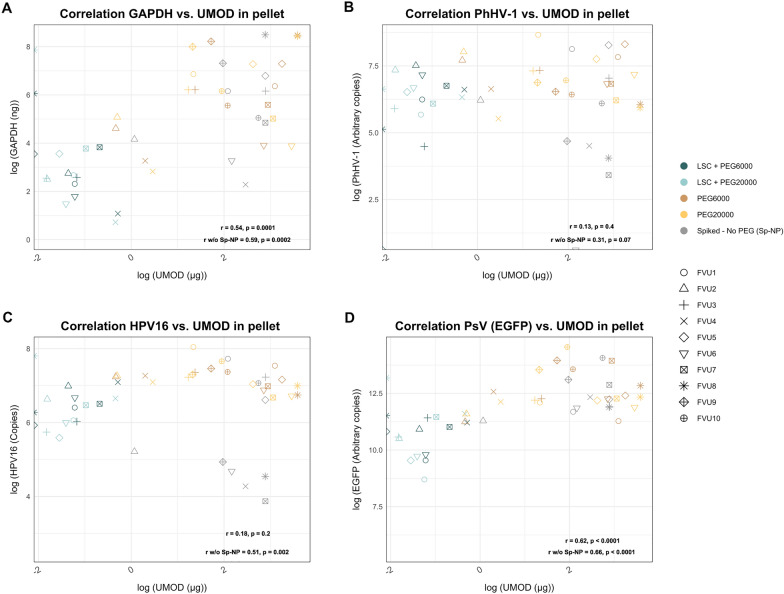
Correlation plots between the amount of **A** GAPDH; **B** PhHV-1; **C** HPV16; and **D** PsV (EGFP) DNA and UMOD in the pellet. Spearman rank correlation coefficients are presented in the figure with, and without (w/o) inclusion of the Sp-NP arm data

UMOD concentrations were measured for specific arms and baseline samples (Table 1). We observed no significant correlations between the respective DNA biomarker quantity and UMOD in the baseline samples (Additional file 1). Additionally, no significant correlations were observed between the quantity of UMOD at baseline and the amount of pelleted DNA for any of the biomarkers and all arms (*p* ≥ 0.08). However, combining all acquired pellet data on DNA and UMOD quantities, we did observe a significant correlation between the amount of GAPDH (*r*_s_ = 0.54, *p* = 0.0001) and PsV (EGFP) (*r*_s_ = 0.62, *p* < 0.0001) DNA and the amount of UMOD in the pellet (Fig. 4). This was not observed for PhHV-1 or HPV16 DNA (*p* ≥ 0.2). Analyzing only the pellet data of the arms with PEG precipitation, thus excluding the Sp-NP arm, we found significant correlations between the levels of GAPDH (*r*_s_ = 0.59, *p* = 0.0002), HPV16 (*r*_s_ = 0.51, *p* = 0.002), and PsV (EGFP) (*r*_s_ = 0.66, *p* < 0.0001) and the amount of UMOD in the pellet but not for PhHV-1 DNA (*p* = 0.07) (Fig. 4). The median (IQR) amount of UMOD was higher in pellet than in the supernatant for the Sp-NP arm, or for the two PEG arms without low-speed centrifugation (Table 1). However, for the arms with low-speed centrifugation, the remaining UMOD in the sample was higher in the supernatant than in the pellet. The amount of UMOD in the baseline sample significantly correlated with the amount of pelleted UMOD for the PEG arms without LSC (*r*_s_ ≥ 0.82, *p* ≤ 0.006) but not in the Sp-NP or LSC + PEG arms (*r*_s_ ≤ 0.50, *p* ≥ 0.14).

## Discussion

Non-invasive sampling can be the solution to reach a larger population for screening, epidemiological trials, and vaccine studies, among others. An example of a non-invasive sample of the urogenital tract is FVU. Secretions of the uterine, cervical, and vaginal epithelium accumulate between the labia minora, are washed away during urination, and are concentrated in the first urine void [41]. In the past decades, studies have proven that FVU has vast advantages for HPV-related research, screening, and vaccine follow-up in women [5, 8, 13, 42–44]. Additionally, this non-invasive sample type could play a crucial role in cervical cancer treatment strategies involving immune checkpoint inhibitors, which has also gained interest in the past few years [45, 46]. Different timings of collection, sample volumes, and storage buffers for FVU have been evaluated, and good agreement has been observed with cervical and vaginal samples for virological endpoints and with serum for immunological endpoints [6, 10, 12, 26, 37, 47, 48]. In addition to HPV-related research, FVU samples could be used as a sample for other sexually transmitted infections (STIs) or as a biomarker source for cancer-related research [17–22].

In this study, we aimed to investigate the effect of an external (PEG) and internal (UMOD) FVU precipitating agent on the concentration of spiked viral cfDNA, human cfDNA and gDNA, and pseudovirion DNA. While one of the advantages of FVU sampling is the concentration of biomarkers of interest related to FGT secretions, other FGT impurities are also concentrated in the sample. These impurities might influence further downstream processing of the sample for future detection of possible biomarkers. As PEG is a known precipitating agent, we evaluated the effect of adding PEG to a FVU sample on the precipitation of cfDNA, gDNA and pseudovirion DNA. It is generally known that cfDNA remains in the supernatant while cellular gDNA precipitates [27, 28]. Earlier studies have shown that both transrenal and locally shed DNA are present in FVU [20, 22]. In HPV-related research for cervical cancer screening, the most important biomarker source is cellular gDNA originating from exfoliated cells of the cervix [49].

Looking at the results of our study, we observed a median 23% increase in precipitated gDNA (GAPDH) when the samples were incubated with PEG6000 before centrifugation and a 55% increase for PEG20000. Additionally, we found a correlation between the amount of UMOD in the pellet and the amount of GAPDH DNA in the pellet for the PEG arms. This correlation increased when the samples without PEG addition (Sp-NP) were excluded from analysis, suggesting that there might be some synergy between the external precipitating agent PEG and the UMOD present in the urine. Since we did not observe a correlation between UMOD in the baseline samples and the precipitation of biomarkers, we hypothesize that the interaction between PEG and the precipitation of biomarkers is influenced not by the amount but by the structure and polymerization of UMOD [50, 51].

The effect of precipitating agents on cfDNA is of great interest to investigate circulating tumor DNA (ctDNA), which is the tumor fraction of cfDNA. Therefore, we also evaluated various precipitation protocols on spiked PhHV-1 and HPV16 plasmid DNA. As cfDNA mostly remains in the supernatant, the influence of a precipitating agent on the location of the DNA is of interest. Here, we also checked whether depleting cellular DNA by a low-speed centrifugation step influenced the results. For both cfDNA markers, PEG addition indeed had a significant effect on precipitation, with median increases of 235% and 329% in pelleted PhHV-1 DNA, and of 773% and 696% in HPV16 plasmid DNA when the samples were incubated with PEG6000 and PEG20000 before centrifugation, respectively. These results clearly show the potential of the precipitating agent PEG on the concentration of cfDNA in FVU and therefore can be of great interest for novel research towards ctDNA in non-invasive urine samples. By adding a low-speed centrifugation step, we observed that 89% of the GAPDH and 41% of the PsV (EGFP) DNA was pelleted, while only 9% of the PhHV-1 and 7% of the HPV16 DNA were pelleted. This clearly shows the depletion of pseudovirion and cellular DNA after low-speed centrifugation. Performing PEG precipitation after low-speed centrifugation caused pelleting of 73% to 85% of the cfDNA but the amount of cfDNA in the pellet was lower (approximately 50%) when no low-speed centrifugation was performed before PEG precipitation. If a clean cfDNA sample is needed, adding this low-speed centrifugation step can be advantageous, although this step can reduce the cfDNA yield. For cell-free plasmid DNA (HPV16), we observed a correlation between UMOD and the pelleted DNA when PEG was added to the samples. This was, however, not observed for the PhHV-1 DNA. Although we did observe a significant correlation between pelleted PhHV-1 and HPV16 DNA for the Sp-NP and PEG6000 arms (*r*_s_ ≥ 0.82, *p* ≤ 0.006), there were differences in precipitation between the PhHV-1 and HPV16 plasmid DNA. These differences may be attributed to variations in DNA fragment size, where the presence of UMOD and PEG enhances DNA precipitation starting from a specific fragment size while having minor effects on smaller DNA fractions.

Another interesting biomarker that was investigated in this study was the DNA of HPV PsV, which was quantified by detecting the DNA of the included reporter (EGFP). It is hypothesized that HPV virions are present in FVU samples although this is expected at low concentrations [14]. To be able to adequately detect virions and eventually use them to investigate infections, they need to be concentrated. In this study, we mimicked the presence of virus particles in FVU by spiking samples with HPV16 PsV. Our results clearly showed that pseudovirions pelleted during centrifugation. During low-speed centrifugation, 59% of the PsV (EGFP) DNA was present in the pellet, and when the sample was centrifuged for 1 h at a faster speed, 90% of the PsV (EGFP) DNA was present in the pellet. We observed a slight increase in the amount of DNA in the pellet when PEG was added as a precipitating agent (99%); however, this change was not significant. A low-speed centrifugation step, to deplete cellular debris and sample impurities is, in this case, not advantageous as 59% of the pseudovirions will be lost. The amount of UMOD in the pellet also correlated significantly with the amount of PsV (EGFP) DNA in the pellet for both the precipitation arms with and without PEG.

An additional and important observation in this study was the difference in the effect of protocols among FVU samples. Each FVU sample has a different composition which also affects the concentration of biomarkers. When examining the samples individually, there was a large variation in the presence of spiked DNA in the pellet or supernatant. Although we spiked all the samples with the same amount of PhHV-1 DNA, HPV16 plasmid DNA, or HPV16 PsV, we did not observe the same trend in concentration for each sample. Therefore, it is essential to always include a minimal amount of samples while optimizing protocols. The UCM:dPBS control supports the fact that the urine sample composition affects the precipitation of biomarkers. For cfDNA, the control sample results closely aligned with the median values observed in the pellet arms. However, when examining PsV (EGFP), urine composition had a clear positive impact on precipitation as the quantities were the lowest for this control sample.

This study has certain limitations. The first limitation is the limited sample size of this study. However, the results clearly show heterogeneity among the different FVU samples while also showing significant effects of the various protocols, ensuring that the sample size is sufficient to support the interpretation of our results. Another limitation is the absence of true standard curves for PhHV-1 and PsV (EGFP) DNA. This limitation also had minimal effect on the data, as the goal of this study was to assess the effect of several protocols and precipitating agents on the presence of DNA. Additionally, spiking the FVU samples with PsV mimics the presence of HPV wild-type virions but is not identical since virions are not only freely present as particles but also encapsulated within cells. Furthermore, extrapolation to urine collected in a different way or without buffer needs to be handled carefully as the storage and collection of the sample might influence the results. The buffer used aids in adjusting the urine sample to a near-neutral pH (between 6.04 and 6.95), which is required for PEG to precipitate and contains salt, known to enhance PEG precipitation [52]. Evaluating unbuffered urine samples may lead to different results.

## Conclusion

In summary, this study presents compelling data regarding the effect of precipitation agents and protocols on the pelleting of cfDNA, cellular gDNA, and pseudovirions in non-invasively collected FVU. The results showed that PEG has a clear positive effect on the concentration of DNA in the pellet and that the effect was largest for cfDNA. A low-speed centrifugation step might also be helpful for depleting FVU of gDNA, pseudovirions, and other impurities when cfDNA is of interest. Additionally, the results showed that there might be an interaction between UMOD present in FVU and the added precipitating agent PEG, but the amount of UMOD in the baseline sample did not influence the concentration of biomarkers. The results of this study will help researchers choose and optimize protocols for biomarker DNA precipitation.

### Supplementary Information

**Additional file 1: Figure S1.** Histogram representing the amount of **A** GAPDH; **B** PhHV-1; **C** HPV16; and **D** PsV (EGFP) DNA in specific arms and fractions on the left y-axis and the amount of UMOD in the baseline sample (red line).

## Data Availability

The data that support the findings of this study are available from the corresponding author upon reasonable request.

## Abbreviations

cfDNA: Cell-free DNA
EGFP: Enhanced green fluorescent protein
FGT: Female genital tract
FVU: First-void urine
GAPDH: Glyceraldehyde-3-phosphate dehydrogenase
gDNA: Genomic DNA
HPV: Human papillomavirus
LSC: Low-speed centrifugation
PEG: Polyethylene glycol
PhHV-1: Seal herpesvirus 1
PsV: Pseudovirion
qPCR: Quantitative PCR
STI: Sexually transmitted infection
THP: Tamm–Horsfall protein
UCM: Urine conservation medium
UMOD: Uromodulin
