# Supplementary material for: Concentration strategies for spiked and naturally present biomarkers in non-invasively collected first-void urine
Source: Eur J Med Res. 2024 Feb 17;29:131. doi: 10.1186/s40001-024-01719-5 (PMC10873940; doi:10.1186/s40001-024-01719-5)
Supplement: Supplementary file 1 — Additional file 1: Figure S1. Histogram representing the amount of A GAPDH; B PhHV-1; C HPV16; and D PsV (EGFP) DNA in specific arms and fractions on the left y-axis and the amount of UMOD in the baseline sample (red line). [file 40001_2024_1719_MOESM1_ESM.docx]

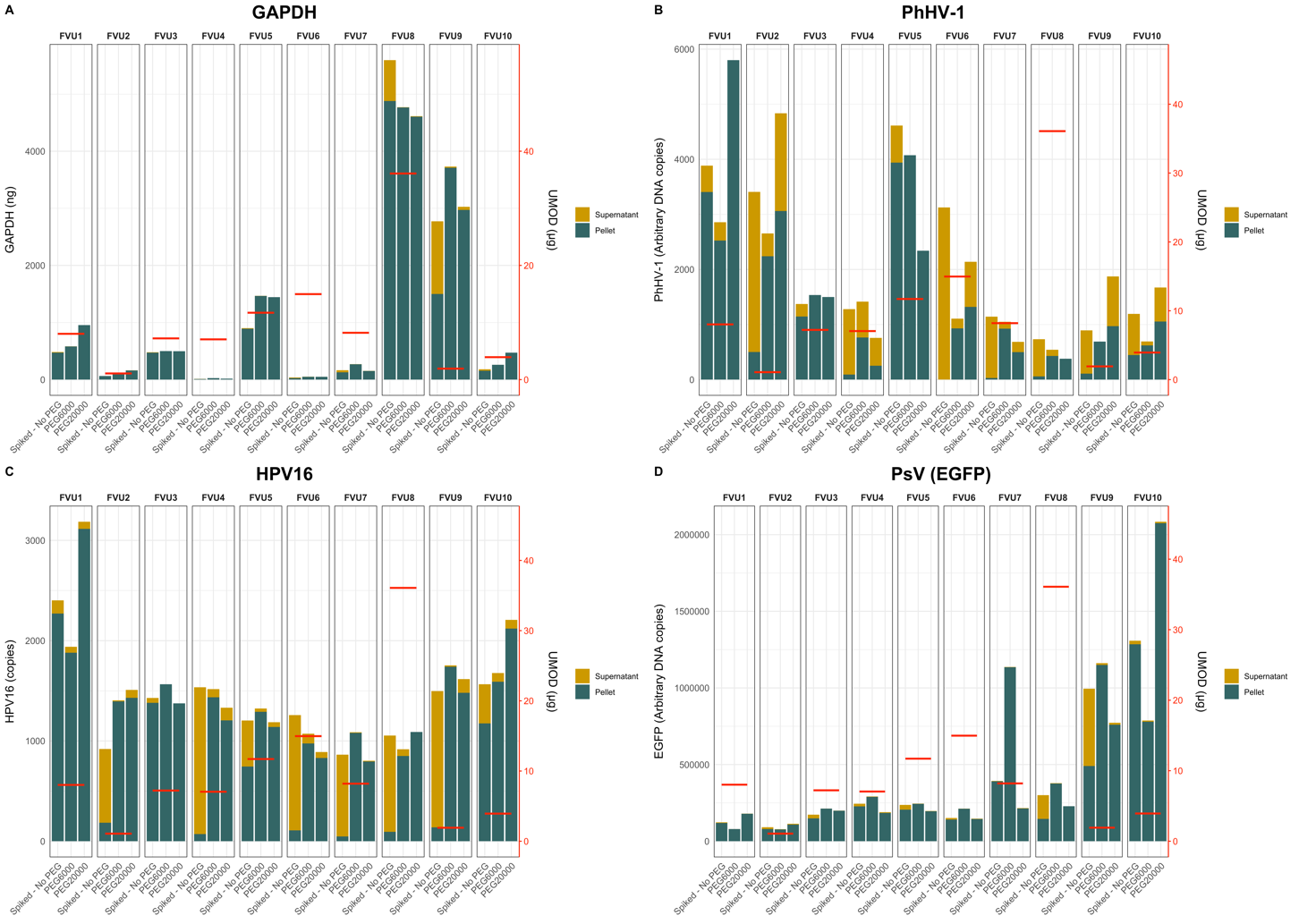

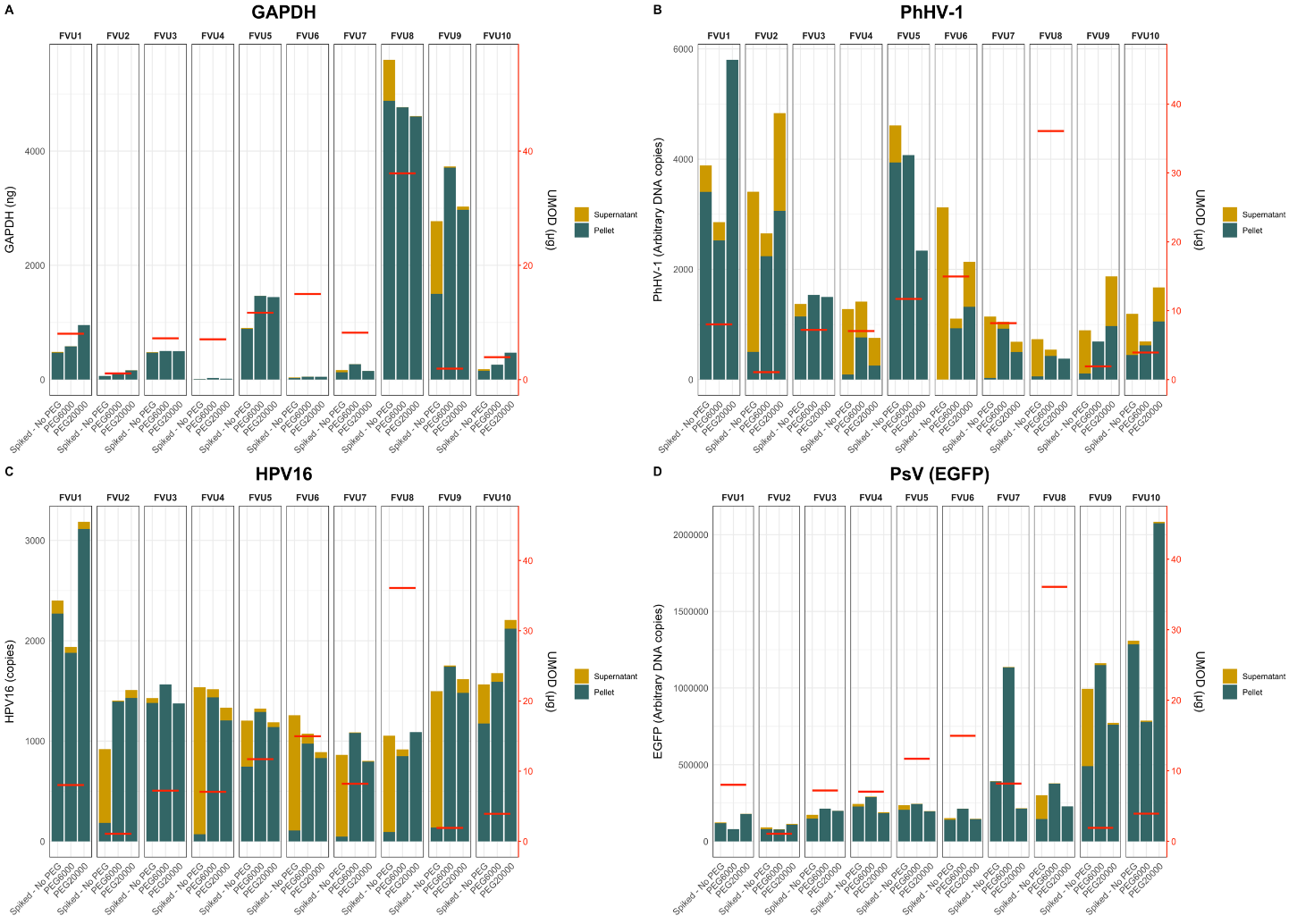

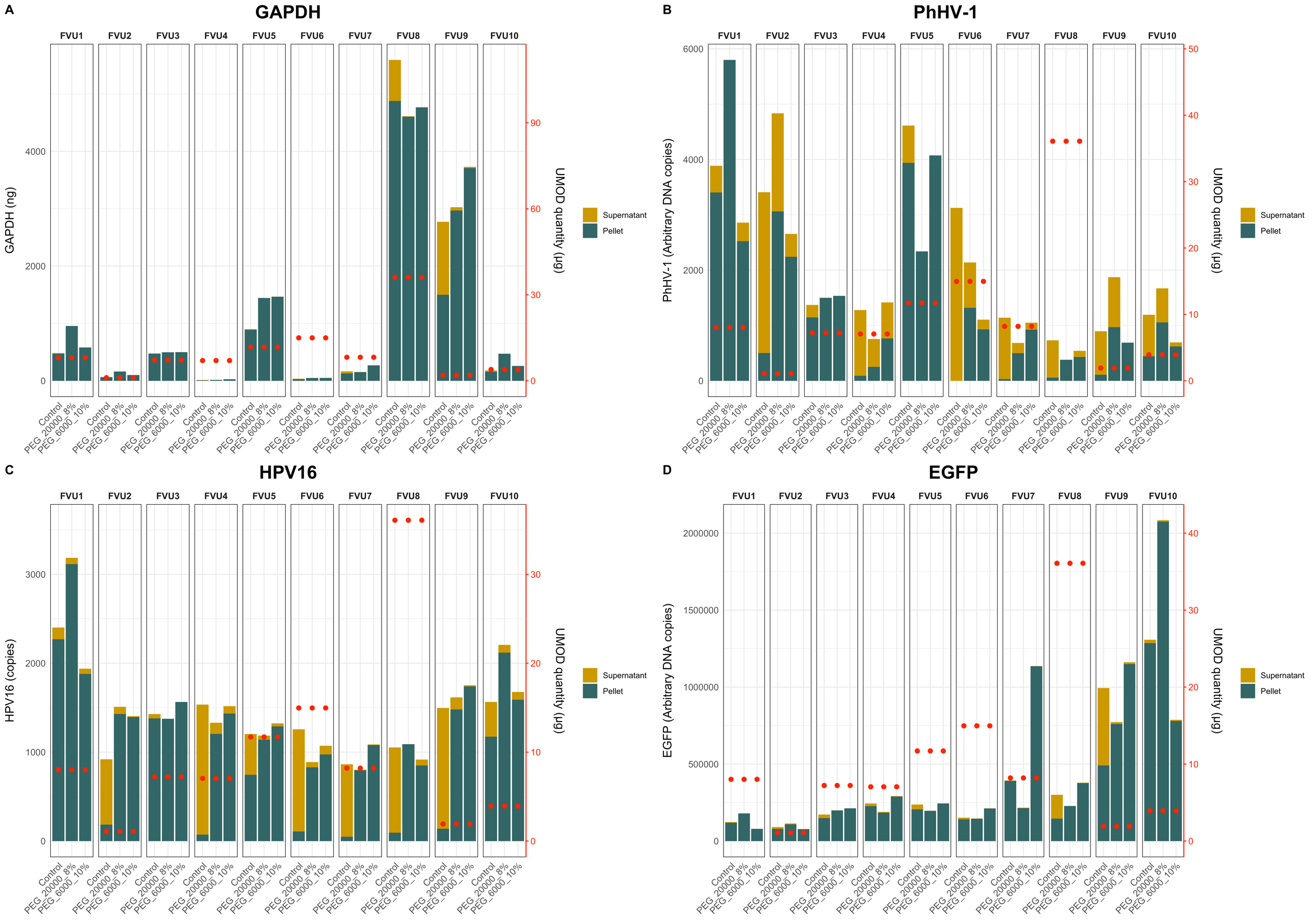


**Additional file 1.** Histogram representing the amount of A) GAPDH; B) PhHV-1; C) HPV16; and D) PsV (EGFP) DNA in specific arms and fractions on the left y-axis and the amount of UMOD in the baseline sample (red line).
